# Mapping the Genetic Relatedness of Outdoor-Biting *Anopheles* Mosquitoes in Zambia

**DOI:** 10.3390/insects16121198

**Published:** 2025-11-25

**Authors:** Reneé L. M. N. Ali, Mary E. Gebhardt, Limonty Simubali, Kochelani Saili, Westone Hamwata, Hunter Chilusu, Mbanga Muleba, Conor J. McMeniman, Anne C. Martin, William J. Moss, Douglas E. Norris

**Affiliations:** 1 The W. Harry Feinstone Department of Molecular Microbiology and Immunology, Johns Hopkins Bloomberg School of Public Health, Baltimore, MD 21205, USA; mgebhar3@jhmi.edu (M.E.G.); cmcmeni1@jhu.edu (C.J.M.); douglas.norris@jhu.edu (D.E.N.); 2 The Johns Hopkins Malaria Research Institute, Johns Hopkins Bloomberg School of Public Health, Baltimore, MD 21205, USAwmoss1@jhu.edu (W.J.M.); 3 Macha Research Trust, Choma P.O. Box 630166, Zambia; limonty.simubali@macharesearch.org (L.S.); kochelani.saili@macharesearch.org (K.S.); 4 National Health Research and Training Institute, Ndola P.O. Box 71769, Zambia; phamwata@gmail.com (W.H.); huntergeorgechilusu@gmail.com (H.C.); mbangamuleba@gmail.com (M.M.); 5 Department of Epidemiology, Johns Hopkins Bloomberg School of Public Health, Baltimore, MD 21205, USA

**Keywords:** outdoor biting, mitochondrial genome, malaria, *Anopheles*, understudied

## Abstract

The zoophilic and exophilic traits of outdoor-biting *Anopheles* have led to this group largely being overlooked for their role in malaria transmission, despite several species now recognized as locally important in regions of sub-Saharan Africa. Given the current limitations with identification of these understudied species, it is crucial to accurately correlate morphological features to molecular data. Here, we produced high quality reference sequence data for representative understudied anopheline species to better understand the phylogenetic relationships between under- and well-studied vectors of malaria. For mitochondrial genome assembly, shallow shotgun sequencing was implemented on single mosquito specimens and phylogenetic analyses were performed on the concatenated protein coding genes of the mitogenomes using a Bayesian approach. This study generated 10 complete mitogenomes focusing on less studied taxa with an average length 15,380 bp and A-T content of 77.4% consistent with other anophelines containing 37 genes. Bayesian inference analysis yielded four main clades with molecular dating indicating that well-studied malaria vectors diverged from outdoor-biting species more than 63 million years ago. These findings support the taxonomic grouping of mosquitoes belonging to the *Anopheles* genus based on morphological characteristics and can provide molecular diagnostics for species identification enabling more precise and adept interventions for malaria control.

## Simple Summary

Outdoor-biting *Anopheles* mosquitoes have been gaining attention due to their potential role in sustaining malaria transmission by avoiding indoor vector control interventions. The efficacy of mitigation efforts that primarily target indoor biting and resting mosquitoes may be undermined by these mosquitoes. The identification of these less studied mosquito taxa is challenging due to cryptic morphological features and the limited number of molecular reference sequences in databases. Advancements in sequencing technologies have led to a steady increase in the generation of mitochondrial genomes (mitogenomes). Mitogenomes have proved to be robust in resolving species identification, population structure and phylogenies in metazoans when compared to commonly used molecular barcodes. Our work highlights the use of mitochondrial genomes for understanding the genetic relatedness of the less-studied outdoor-biting anopheles with reference to the primary vectors of malaria. The datasets generated in this study can be used to improve interventions for malaria control and employ molecular diagnostics for accurate species identification.

## 1. Introduction

As African nations strive towards malaria control and elimination, insecticide resistance and residual malaria transmission challenge current vector-targeted malaria interventions [1,2,3,4]. While the primary vectors *Anopheles funestus*, *An. coluzzii*, and *An. gambiae* are the focus of these mitigation strategies [5,6], selection pressure from indoor-focused measures such as indoor residual spraying (IRS) and insecticide-treated nets (ITNs) have led to changes in mosquito behavior and populations [7,8,9]. One key change is the recognition that secondary *Anopheles* vectors such as *An. rufipes* [10,11], *An. pharoensis* [12], *An. squamosus* [13], and *An. coustani* [13,14,15] contribute to sustaining residual transmission in sub-Saharan Africa. These mosquito species are considered largely exophagic and exophilic, behaviors that have allowed them to evade indoor vector control interventions [7,8,16,17].

Despite frequent collection alongside the primary malaria vectors, the genomics, ecology, biology, and behavior of these long-overlooked anopheline mosquito species are poorly understood [9]. Furthermore, the constraints of overlapping and cryptic morphological features [18,19,20,21], together with the absence of available molecular data in genomic databases [17,20,22,23], have made robust morphological and molecular identification of these less studied anopheline species extremely challenging. Studies have also reported evidence of functional heterogeneity in anopheline genomes which influence their behavioral plasticity, a crucial characteristic for defining vectorial capacity and adaptability [24,25]. Therefore, the accurate identification and bionomic characterization of understudied *Anopheles* species is now critical given their key role as local vectors in driving residual malaria transmission in Zambia, Madagascar, southern Mozambique, Ethiopia, and Kenya [13,15,26,27,28,29].

Although there is an extensive list of *Anopheles* sequences generated using molecular barcodes based on the cytochrome oxidase I (COI) and internal transcribed region 2 (ITS2) genes [12,17,18,20,26,27,28,30,31,32,33,34,35], there still remain limitations in available sequence for these understudied *Anopheles* species to produce robust differentiation between members of closely related taxa [17,18,31,36]. This includes cryptic species that may be incriminated in residual malaria transmission but have been allotted placeholder names such as *An. species 11* [17,18,20], *An. species 15* [18], and *An. species* unknown group 1 [20], particularly in the absence of comprehensive morphological identification to complement the generated molecular barcode sequences. Furthermore, the use of the single COI gene to validate identification for less-studied *Anopheles* has produced matches with low similarities (less than 80%) and weakly supported phylogenies. For instance, this has led to inconclusive identities for members of the *An. coustani* group in earlier studies from Zambia [17,31] and Mozambique [26]. Recently, mitochondrial genomes were used to provide conclusive identities and differentiate the cryptic taxa of the *An. coustani* group into phylogenetically well-supported taxonomic clades [37].

The acquisition of genomic datasets has become more accessible due to the expansion of sequencing and computational technologies, including mitochondrial genomes (mitogenomes), which have shown to be useful in the identification and resolution of phylogenies for several mosquito species and species groups [38,39,40]. These circular, double-stranded DNA molecules encode 37 genes, including 13 protein-coding genes (PCGs), 22 transfer RNA (tRNA), 2 ribosomal RNA (rRNA), and a non-coding control region [40]. In addition to the 13 PCGs, key characteristics such as low incidence of recombination, high copy number, and maternal inheritance make the mitogenome a more effective taxonomic tool compared to single barcodes [40,41,42,43,44,45]. Expanding mitochondrial genome resources to include less studied mosquito species is essential for accurate species delineation and gaining insights into mosquito ecology and systematics for public health interventions. In this study, we aimed to (i) generate mitochondrial genomes for representative understudied and cryptic mosquito species, and (ii) demonstrate the strength of mitogenomes compared to prior studies that were limited to the COI gene in attempts to resolve phylogenies.

## 2. Materials and Methods

### 2.1. Mosquito Collections

Specimens were collected during routine entomological surveillance in Nchelenge, Zambia in 2023–2024 as part of the Southern and Central Africa International Centers of Excellence for Malaria Research (ICEMR) investigations. Miniature CDC Light Traps (John W. Hock Co., Gainesville, FL, USA), were positioned both indoors and outdoors where people gather in the evening and near animal pens.

### 2.2. DNA Extraction and Sequencing

DNA extractions using a modified extraction method [46] were performed on single mosquito specimens morphologically identified as *An. rufipes*, *An. maculipalpis*, *An. pretoriensis*, *An. squamosus*, and *An. pharoensis* [21]. The extracted DNA and previously extracted specimens identified using the COI gene as species 11, species 15, unknown group 1, unknown group 2, and unknown group 3 from a previous study [20] were quantified using the Qubit dsDNA assay kit (Thermo Fisher Scientific, Waltham, MA, USA) and shipped to SeqCenter (Pittsburgh, PA, USA) for library construction and Illumina sequencing. The libraries were 150 bp paired end sequenced to a count of 13.3 million reads per sample.

### 2.3. Mitochondrial Genome Assembly and Annotation

The mitochondrial genomes were assembled using NOVOPlasty [47] (RRID:SCR_017335) version 4.3.5 with k-mer set at 39 base pairs and *An. squamosus* (OP_77691) as the seed sequence. Using the MITOchondrial genome annotation (MITOS) [47] galaxy tool, generated contigs were automatically annotated using the invertebrate genetic code under default settings. The start and stop codon positions of the annotated contigs were manually adjusted in Geneious Prime (RRID:SCR_010519) version 2025.1.2 (Biomatters, Auckland, New Zealand) using reference anopheline mitochondrial genomes as a guideline. The generated contigs with corresponding annotations were submitted to the GenBank database for the assignment of accession numbers.

### 2.4. Phylogenetic Analysis and Tree Construction

Using jModelTest (v2.1.10) [48], the best fit base pair substitution model based on the Akaike information criterion (AIC) and the Bayesian information criterion (BIC) was determined under default settings using an aligned sequence matrix. This alignment was generated using the MAFFT alignment tool implemented in the Geneious Prime (RRID:SCR_010519) version 2025.1.2 (Biomatters, Auckland, New Zealand from the 13 concatenated PCGs of mitogenomes generated in this study, available mitochondrial genomes of understudied African anopheline species; *An. marshallii* (NC_064607), *An. moucheti* (NC_064608), *An. gibbinsi* (OR_569715), *An. nili* (NC_064610), *An. squamosus* (OP_776919, PP_093769), *An. pharoensis* (PP_105075), *An. pretoriensis* (PP_068258, PP_105074), *An. maculipalpis* (NC_064606, PP_093768), *An. rufipes* (PP_105076), and *An. coustani* group (PQ_585798, PQ_587039, PQ_587041, PQ_587036, PP_375116), and reference mitogenome sequences for the well-studied species *An. gambiae* (MG_930894), *An. arabiensis* (NC_028212), and the *An. funestus* group (MG_742172, MG_742194, MT_917162, MT_917137, MT_91714, MT_917157, MT_917163). Using Bayesian Evolutionary Analysis by Sampling Trees (BEAST) 2 software [49], inference analysis was performed on the aligned sequence matrix using tree independent runs and a 20% burn-in rate for tree building purposes under default settings. Bayesian analysis was also performed on an alignment generated from COI sequences available from GenBank complementary to representative species in the mitochondrial genome tree. Trees were visualized and annotated using FigTree v.1.4.4 (Available online: http://tree.bio.ed.ac.uk/software/figtree/ (accessed on 29 October 2025).

### 2.5. Dating Time Estimation

Divergence time estimations were calculated using the previously mentioned parameters used for Bayesian inference in BEAST2. The frequently referenced Aedes–Anopheles divergence time of approximately 154.7 million years ago (MYA) [50] was used as the calibration point set to normal distribution.

## 3. Results

The 13 mitochondrial genomes produced in this study were consistent with other African anopheline mitogenomes represented in NCBI’s GenBank database, which comprised 13 PCGs, 22 tRNAs, and 2 rRNAs, with lengths ranging from 15,534 bp (Unknown group 2) to 15,346 bp (*An. pharoensis*) and a mean AT content of 77.4% (Table 1).

**Figure 1:**
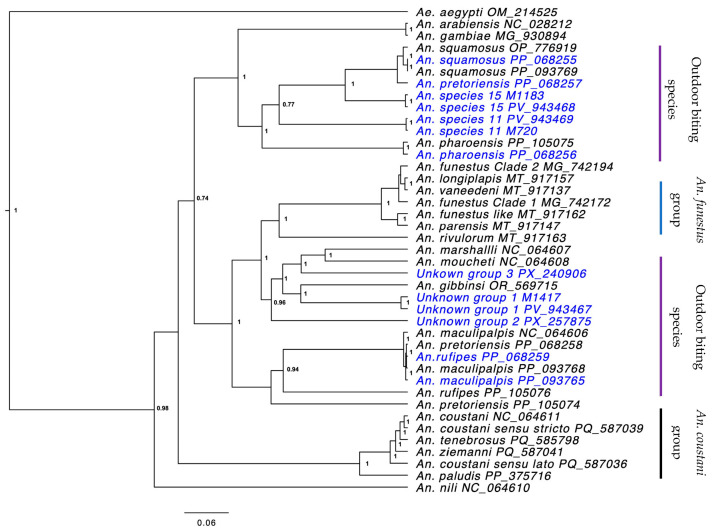
Bayesian tree showing the phylogenetic relationship of 13 new mitochondrial genomes (highlighted in blue) of understudied *Anopheles* mosquito species with other available anopheline sequences. The tree includes assigned accession numbers and was constructed using BEAST v2.7.6. The posterior probabilities supporting the tree topology are represented by the values at the nodes.

**Figure 2:**
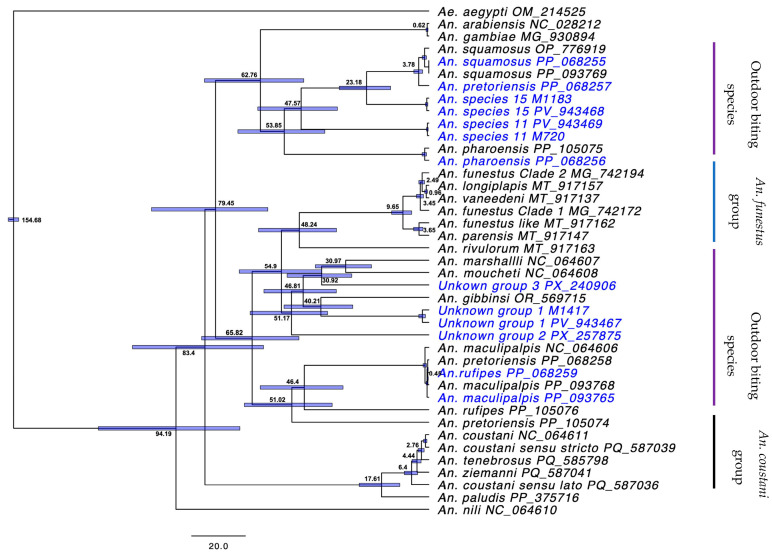
Phylogenetic tree showing inferred molecular divergence estimates (MYA) for outdoor-biting *Anopheles* using the concatenated PCGs from mitogenomes generated in this study. The mean divergence time (MYA) predicted for each event is represented by the values at the tree nodes. The bars show the values at the tree nodes, 95% confidence intervals.

Bayesian inference for the mitochondrial genomes resulted in a phylogenetic tree that separated specimen sequences (Figure 1) compared to COI tree which resulted in 4 weakly supported main clades (Figure S1). The most recent common ancestor (MRCA) for *An. funestus* and *An. gambiae*, with the outdoor-biting *Anopheles* included in this study, dated back to 54.9 and 62.76 MYA, respectively (Figure 2).

## 4. Discussion

Bayesian phylogenetic analysis based on the concatenated PCGs from the mitogenomes was able to clearly delineate less-studied African anopheline taxa compared to phylogenetic analyses using exclusively the COI gene as in earlier studies [17,20]. Several of these species are often misidentified during morphological examination or matched to an unassigned species following molecular barcoding [20,26,33]. This may be attributed at least in part to the unknown diversity of outdoor-biting anophelines and relative inexperience in morphological identification of these numerous taxa, some of which may yet to be fully described due to the bias placed on understanding highly anthropophilic, endophagic, and endophilic species [6,9,17,28,31]. The uncertainty of vector richness of the exophagic anophelines, together with their perceived adaptability and undetermined foraging times, habitats, and opportunistic feeding patterns, have led to ambiguous species assignments [9,14,28,33,51]. This combination of characteristics emphasizes the need for the expansion of outdoor surveillance and investigation of the anophelines found in this space, as well as innovative strategies to overcome the shortcomings of indoor-focused interventions.

A growing number of understudied anophelines have been confirmed or implicated in human malaria transmission. Despite historic consideration as a secondary vector [12,17,27,52], *An. coustani* is now regarded as a primary local vector in regions of Madagascar [15,29,53]. Additionally, previous studies have identified *An. pretoriensis* [34], *An. pharoensis* [52,54], and cryptic anopheline species [17,18,20] demonstrating opportunistic feeding patterns on humans, some infected with sporozoites of human malaria parasite species. Anopheles squamosus is another species strongly implicated as a malaria vector, with a wide geographical range, and has demonstrated variable foraging behavior towards human blood meals [13,55,56]. Related to this are a number of ‘molecular taxa’. Examples include *An. species 11* and *An. species 15*, which are often morphologically keyed as *An. squamosus* but are differentiated by the COI barcode and even more strongly by the mitogenome sequence (Figure 1) [17,20]. Others include *An. unknown groups 1–3* for which morphology and molecular barcoding was inconclusive [20]. Here, the mitogenome data provide the most comprehensive insight into the taxonomic placement of these ‘molecular taxa’, but as with prior studies, without a more extensive sequence database of recognized species, these specimens remain taxonomically unresolved. The fact that many of these exophagic taxa cluster together in the phylogenetic analysis may be an artifact of their shared ancestry and that these share behavioral adaptations may have been reinforced over millennia.

It is clear that full mitogenomes offer much more discriminatory power for a phylogenetic approach to inquire about shared biological traits and possibly ascertain whether behaviors such as biting preference are due to recent adaptations or reflect the existence of genetically distinct lineages which may have been overlooked when restricted to morphological identification. Dating time estimations from well-recognized malaria vectors further corroborate the presence of these outdoor-biting *Anopheles* as cryptic lineages with distinct ecological niches, suggestive of understudied species that may maintain transmission outdoors, perhaps under certain conditions such as relative absence of non-human hosts, or human behavior that promotes high opportunistic human-biting rates. Furthermore, the accurate taxonomic placement of these mosquitoes highlights the relationships between known vectors and putative vector species which may provide further insights into understanding the differences in biting, foraging, and vectorial capacity of these less-studied species. Linking morphological reference specimens to genomic data is key for the accurate identification given the status of unassigned anopheles species with sporozoites collected in the field [18,19,26,52].

## 5. Conclusions

Although reference sequences are available for many commonly encountered outdoor-biting anopheline species, there remains a paucity of data to accurately identify and taxonomically place these species in the wider *Anopheles* genus. This study contributes valuable genetic datasets representing exophagic species collected in Zambia and present across the African continent. The generation of mitochondrial genomes for cryptic unassigned species that are commonly collected has given priority to the use of integrative taxonomy in future research. The linking of molecular data with morphological and type specimens can further strengthen the credibility of species delimitation for the assigned zoological nomenclature of these cryptic taxa. The analyses from this study identified the phylogenetic relationships between the primary malaria vectors and understudied species implicated in malaria transmission, assisting to close the genetic gap of what we know about these anophelines of public health importance.

## Figures and Tables

**Table 1:** Genome characteristics for mitochondrial genomes of 13 understudied anopheline mosquito species generated in this study.

Identification	Contig Size	GC%	AT%	GenBank Accession
Morphological				
*An. pretoriensis*	15,348	23.0	77.0	PP_068257
*An. pharoensis*	15,346	23.7	76.3	PP_068256
*An. rufipes*	15,362	22.9	77.1	PP_068259
*An. squamosus*	15,349	23.1	76.9	PP_068255
*An. maculipalpis*	15,361	23.4	76.6	PP_093765
Molecular				
*An. species 11* *An. species 11*	15,354 15,350	23.0 23.1	77.0 76.9	PV_943469 PX_583105
*An. species 15* *An. species 15*	15,350 15,354	20.0 22.8	80.0 77.2	PV_943468 PX_583106
Unknown group 1 Unknown group 1	15,398 15,394	22.5 22.1	77.5 78.9	PV_943467 PX_583104
Unknown group 2	15,534	23.1	76.9	PX_257875
Unknown group 3	15,436	20.3	79.7	PX_240906

## Data Availability

The mitochondrial genomes are available with accession numbers PP068255–PP068257, PP068259, PP093765, PV943467–PV943469, PX240906, and PX257875.

## Supplementary Materials

The following supporting information can be downloaded at https://www.mdpi.com/article/10.3390/insects16121198/s1. Figure S1: Bayesian tree showing the phylogenetic relationship of less studied *Anopheles* mosquito species with main vectors on malaria using the cytochrome oxidase I gene (COI). The posterior probabilities supporting the tree topology are represented by the values at the nodes.

## Abbreviations

The following abbreviations are used in this manuscript:

COI	Cytochrome Oxidase I
ITS2	Internal transcribed spacer 2 region
PCGs	Protein coding genes
tRNA	Transfer RNA
rRNA	Ribosomal RNA
ICEMR	International Centers of Excellence for Malaria Research
AIC	Akaike information criterion
BIC	Bayesian information criterion
BEAST	Bayesian Evolutionary Analysis by Sampling Trees
MYA	Million years ago
