# Supplementary material for: Mapping the Genetic Relatedness of Outdoor-Biting Anopheles Mosquitoes in Zambia
Source: Insects. 2025 Nov 25;16(12):1198. doi: 10.3390/insects16121198 (PMC12734120; doi:10.3390/insects16121198)
Supplement: Supplementary file 1 [file insects-16-01198-s001.zip › insects-3920048-supplementary.pdf]

Supplementary data for:

Mapping the Genetic Relatedness of Outdoor-Biting *Anopheles* mosquitoes in Zambia

Reneé L.M.N. Ali[1,2], Mary E. Gebhardt[1,2], Limonty Simubali[3], Kochelani Saili[3], Westone Hamwata[4], Hunter Chilusu[4], Mbanga Muleba[4], Conor J. McMeniman[1,2], Anne C. Martin[2,5], William J. Moss[2,5], Douglas E. Norris[1,2]

[1]The W. Harry Feinstone Department of Molecular Microbiology and Immunology, Johns Hopkins Bloomberg School of Public Health, Baltimore, MD, USA.

[2]The Johns Hopkins Malaria Research Institute, Johns Hopkins Bloomberg School of Public Health, Baltimore, MD, USA.

[3]Macha Research Trust, Choma, Zambia.

[4]National Health Research and Training Institute, Ndola, Zambia.

[5]Department of Epidemiology, Johns Hopkins Bloomberg School of Public Health, Baltimore, MD, USA.

Corresponding author: rali23@jh.edu

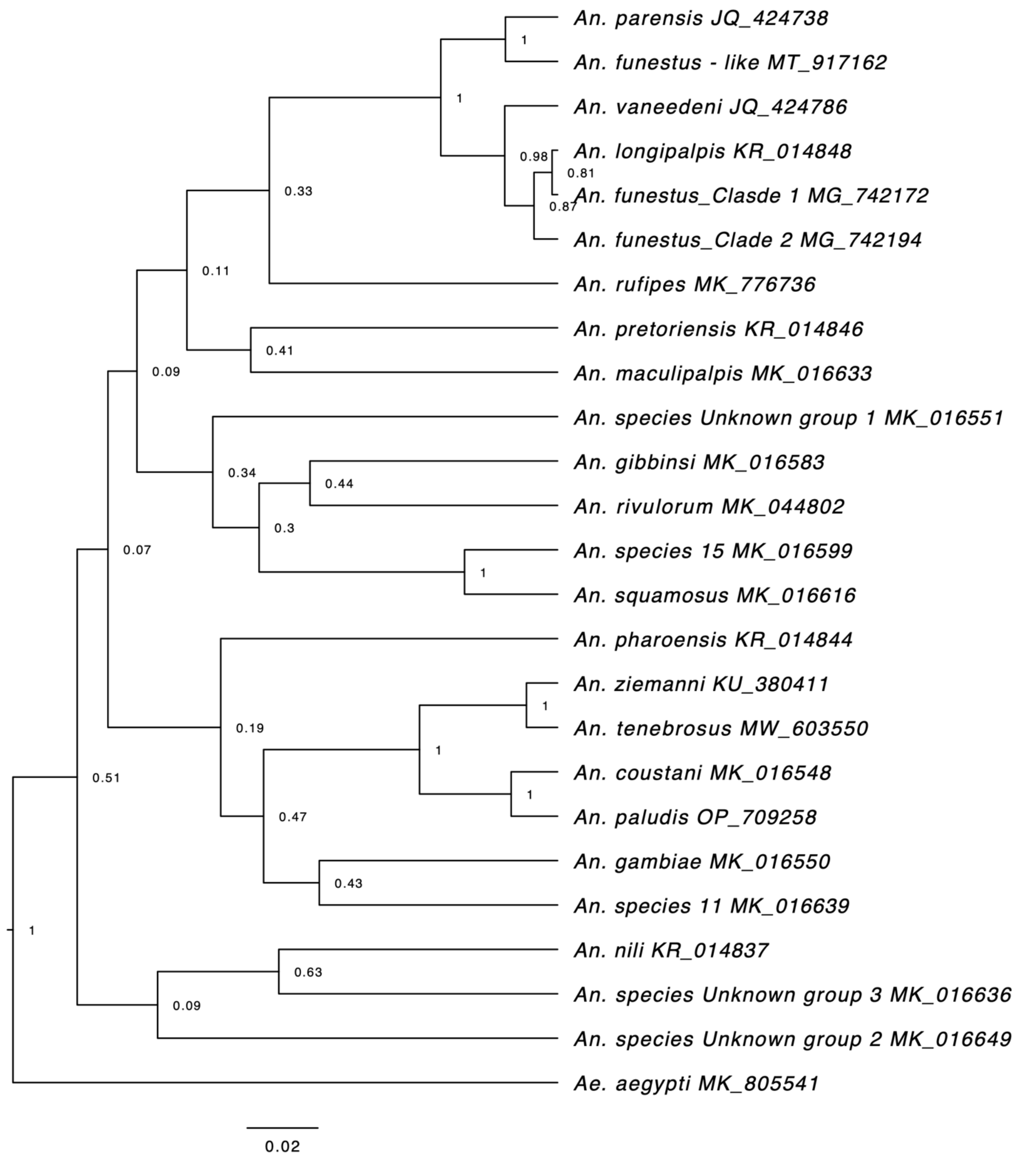

Figure S1. Bayesian tree showing the phylogenetic relationship of less studied *Anopheles* mosquito species with main vectors on malaria using the cytochrome oxidase I gene (COI) using. The posterior probabilities supporting the tree topology are represented by the values at the nodes.
